# TomatoWUR: An annotated dataset of tomato plants to quantitatively evaluate segmentation, skeletonisation, and plant-trait extraction algorithms for 3D plant phenotyping

**DOI:** 10.1016/j.dib.2025.111852

**Published:** 2025-07-07

**Authors:** Bart M. van Marrewijk, Tim van Daalen, Katarína Smoleňová, Bolai Xin, Gerrit Polder, Gert Kootstra

**Affiliations:** a Wageningen University and Research, Wageningen, the Netherlands; b College of Engineering, South China Agricultural University, Guangzhou, China

**Keywords:** Digital plant phenotyping, Tomato plants, Horticulture, Plant architecture, 3D point clouds, RGB images

## Abstract

Plant phenotyping involves the measurements of plant traits to gain more insight into the interaction between the genotype (G), environment (E) and crop management strategies (M). To improve plant phenotyping, accurate measurements are crucial. Manual measurements are biased, time-intensive, and therefore limited to only a few plants. Especially measurements of 3D phenotypic traits, such as plant architecture, internode length, and leaf area are difficult to extract manually. To enhance the speed and accuracy of phenotyping, there is a need for automatic digital plant phenotyping solutions. The presented dataset contains 3D point clouds of tomato plants, which will enable researchers to develop novel methods to extract 3D phenotypic traits. Converting 3D point clouds to plant traits is also known as 3D plant phenotyping. This process can be subdivided into three steps: point cloud segmentation, skeletonisation to extract plant architecture, and plant-traits extraction. Those three steps need to be analysed properly to indicate bottlenecks and improve 3D phenotyping algorithms.

Currently, the development of 3D phenotyping algorithms is inhibited by the availability of comprehensive datasets and algorithms to analyse all steps. To our best knowledge only five annotated datasets exist for testing and validating 3D phenotyping algorithms. However, these datasets mainly focus on the segmentation step. Skeletonisation and manual measured plant traits are frequently not included. To improve 3D plant phenotyping, a novel dataset, TomatoWUR, is presented. This comprehensive dataset consists of 44 point clouds of single tomato plants imaged by fifteen cameras to create a point cloud using the shape-from-silhouette methodology. The dataset includes annotated point clouds, skeletons, and manual reference measurements. In addition, the dataset includes software for comprehensive evaluation and comparison of phenotyping methods, which is expected to benefit the development of 3D phenotyping algorithms. The related software can be found our GIT: https://github.com/WUR-ABE/TomatoWUR.

Specifications Table

[Instruction for Specifications Table in comment box]

**Table:** 

Subject	Biology
Specific subject area	The dataset aims to improve plant phenotyping in 3D.
Type of data	Raw images: PNG Annotated RGB images: PNG Camera calibration: json Point clouds: csv, including coordinates (xyz), colour (r,g,b), normals (nx, ny, nz), Annotated point clouds: csv Annotated skeletons including manual measurements: csv
Data collection	In this research, we created a dataset of 44 annotated 3D point clouds of tomato plants. Images were captured using fifteen cameras surrounding a single plant. Those images were used to create a point cloud using the shape-from silhouette methodology [1]. The resulting dataset was annotated and separated into three parts. 1) Annotated point clouds and corresponding RGB images with semantic and instance labels, 2) annotated skeletons to analyse plant architecture, and 3) manual reference measurements of internode length, internode diameter, leaf angle, and phyllotactic angle to evaluate phenotyping algorithms from point cloud to plant traits.
Data source location	Plants were grown in the greenhouse of the Netherlands Plant Eco-phenotyping Centre (NPEC) located in Wageningen at latitude: 51.989471, longitude: 5.663269. Data is stored at data servers of the Dutch 4TU.Federation foundation (4TU.ResearchData).
Data accessibility	Repository name: TomatoWUR: an annotated dataset of 3D tomato plants to quantitatively evaluate segmentation, skeletonisation, and plant trait extraction algorithms for 3D plant phenotyping Data identification number: 10.4121/e2c59841-4653-45de-a75e-4994b2766a2f Direct URL to data: http://doi.org/10.4121/e2c59841-4653-45de-a75e-4994b2766a2f
Related research article	*A part of the dataset is related to following paper:* *3D plant segmentation: Comparing a 2D-to-3D segmentation method with state-of-the-art 3D segmentation algorithms [**2**].*https://doi.org/10.1016/j.biosystemseng.2025.104147

## 1 Value of the Data

• Plant phenotyping is the study of measuring observable plant traits to better understand the physiology and growth of plants. The phenotype (P) of a plant is influenced by genotype (G), environment (E) and crop management (M). This interaction is also known as P=GxExM. At the moment, methods for digital plant phenotyping are mostly limited to the 2D plane based on images. Plants, however, exhibit important 3D spatial traits that are relevant for phenotyping. Especially the plant architecture, describing how stem, branches, and leaves are connected is of importance because of its influence on the light interception and photosynthesis. Phenotyping in 3D is needed to extract those traits.
• Currently, the number of annotated datasets for 3D phenotyping is limited. To the best of our knowledge, only five annotated point-cloud datasets exist for phenotyping: Rose-X [3], Soybean-MVS [4], Pheno4D [5], Last-Straw [6], PLANesT-3D [7]. The datasets are summarized in Table 1.

**Table 1:** Summary of available datasets for 3D phenotyping.

Dataset	Crop	Annotations (public available)	references
Rose-x	(synthetic) roses	Semantic	[3]
Soybean-MVS	Soybean	Semantic	[4]
PLANesT-3D	Pepper, rose and ribes	Semantic + instances	[7]
Pheno4D	Maize and (small) tomato plants	Semantic + instances + timeseries	[5]
Last-Straw	Strawberries	Semantic + instances + timeseries + individual skeletons	[6]
TomatoWUR	(medium sized) Tomato plants	Semantic + instances + connected skeletons + manual measurements
• Unfortunately, in all published datasets, manual measurements and connected skeletons were not included. To improve digital plant phenotyping, we present TomatoWUR, a comprehensive dataset of point clouds including semantic labels, instances, skeletons and manual measurements.
• 3D plant phenotyping typically consists of three steps: point cloud segmentation, skeletonisation and trait extraction. In this context a skeleton is defined as a set of nodes, connected with edges. Those nodes and edges basically represent the plant architecture. For example, the connection between two nodes can describe internode length or leaf angle. Our comprehensive dataset makes it possible to train and evaluate each individual step. Existing datasets only provide annotations for segmentation. In the TomatoWUR dataset all three steps are annotated. In addition, software is included for each evaluation step to improve standardisation. Therefore, we believe that that the TomatoWUR dataset is an important contribution to the plant phenotyping community. It paves the way to optimize segmentation, skeletonization, and plant trait extraction methods to improve 3D plant phenotyping.
• Step 1 – point-cloud segmentation is crucial as it enables to analyse specific plant traits, for example, estimating leaf fresh weight or identifying the starting point of the main stem, which is required for most skeletonisation algorithms. The TomatoWUR dataset can be used to optimize and validate point cloud segmentation algorithms. The annotated tomato point clouds consist of five classes: 1=leaves, 2=main stem, 3=pole (supporting stick), 4=side stem, representing petiole, petiolule and rachis, 255=not annotated / noise. The distinction of the main, side and pole class makes the dataset interesting for state-of-the-art algorithm because those three classes all belong to circular shaped parts of the plant, which are difficult to segment. Most datasets in literature, like Rose-X, PLANest-3D and Pheno4D, do not classify the side stem as a separate class, thereby simplifying the segmentation task.
• Step 2 - analysing the plant architecture by extracting plant skeletons. The 3D plant architecture describes how stems, branches, and leaves connect to each other spatially. This information is crucial to extract important plant traits, for example, the internode length, leaf angle, and phyllotactic angle can be extracted from skeletons. The plant skeleton is therefore well-suited to represent the 3D plant architecture. In literature, evaluation of skeletonisation methods frequently relies on the indirect comparison between the measured and predicted plant traits [8,9] without considering the reconstruction quality of the skeleton. The TomatoWUR dataset includes annotated skeletons and a novel evaluation methodology to assess the performance of skeletonisation algorithms.
• Step 3 - conversion from skeletons to plant traits. To validate plant measurements, the TomatoWUR dataset contains manual plant-trait measurements of the internode diameter, internode length, leaf angle, and phyllotactic angle. These measurements are linked to the annotated skeletons to fully automate the evaluation of the 3D phenotyping algorithms. TomatoWUR is therefore a comprehensive dataset to fully support the development of 3D plant phenotyping algorithms and evaluate these algorithms in three separate steps: segmentation, skeletonization, and plant trait evaluation.

## 2 Background

As indicated in value of data, accurate phenotyping of plants is essential to find the relation between genotypes, environment, crop management, and the plant phenotype. 3D plant phenotyping focuses on processing point clouds to extract 3D plant traits.

To improve 3D plant phenotyping, more comprehensive open-source datasets are needed including point clouds, skeletons to describe the plant architecture and manual measurements of plant traits. Most datasets such as Rose-X [3], Pheno4D [5], Soybean-MVS [4] and PLANesT-3D [7] only include semantic and instance labels. In the paper of Pheno4D, a comparison was made with manual measurements, but the published dataset did not include skeletons and manual measurements, making optimisation and evaluation of 3D plant phenotyping algorithms challenging.

To evaluate 3D plant phenotyping algorithms, we propose TomatoWUR, a comprehensive dataset including software to evaluate and improve 3D phenotyping pipelines in three steps: 1) Evaluation of semantic segmentation algorithms, 2) evaluation of skeletons to analyse plant architecture, and 3) evaluation of manual measurements. Those three-evaluation metrics can be used to optimise phenotyping algorithms to assist researchers and breeders linking the genotype and environment with crop traits.

## 3 Data Description

### 3.1 Data structure

**Fig. 1:**
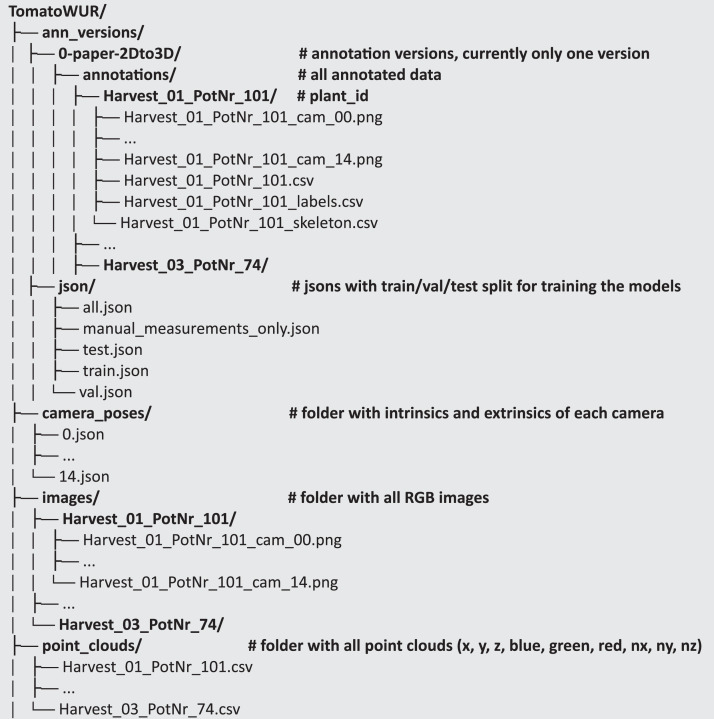
Dataset structure and files

The data can be downloaded from: http://doi.org/10.4121/e2c59841-4653-45de-a75e-4994b2766a2f, containing 3.4Gb. In Fig. 1, the dataset structure is summarised. It contains four folders’ *images, point_clouds, ann_versions and camera_poses*: The dataset contains in total 44 tomato plants. Each folder is explained in more detail in next paragraphs.

### 3.2 Images folder

**Fig. 2:**
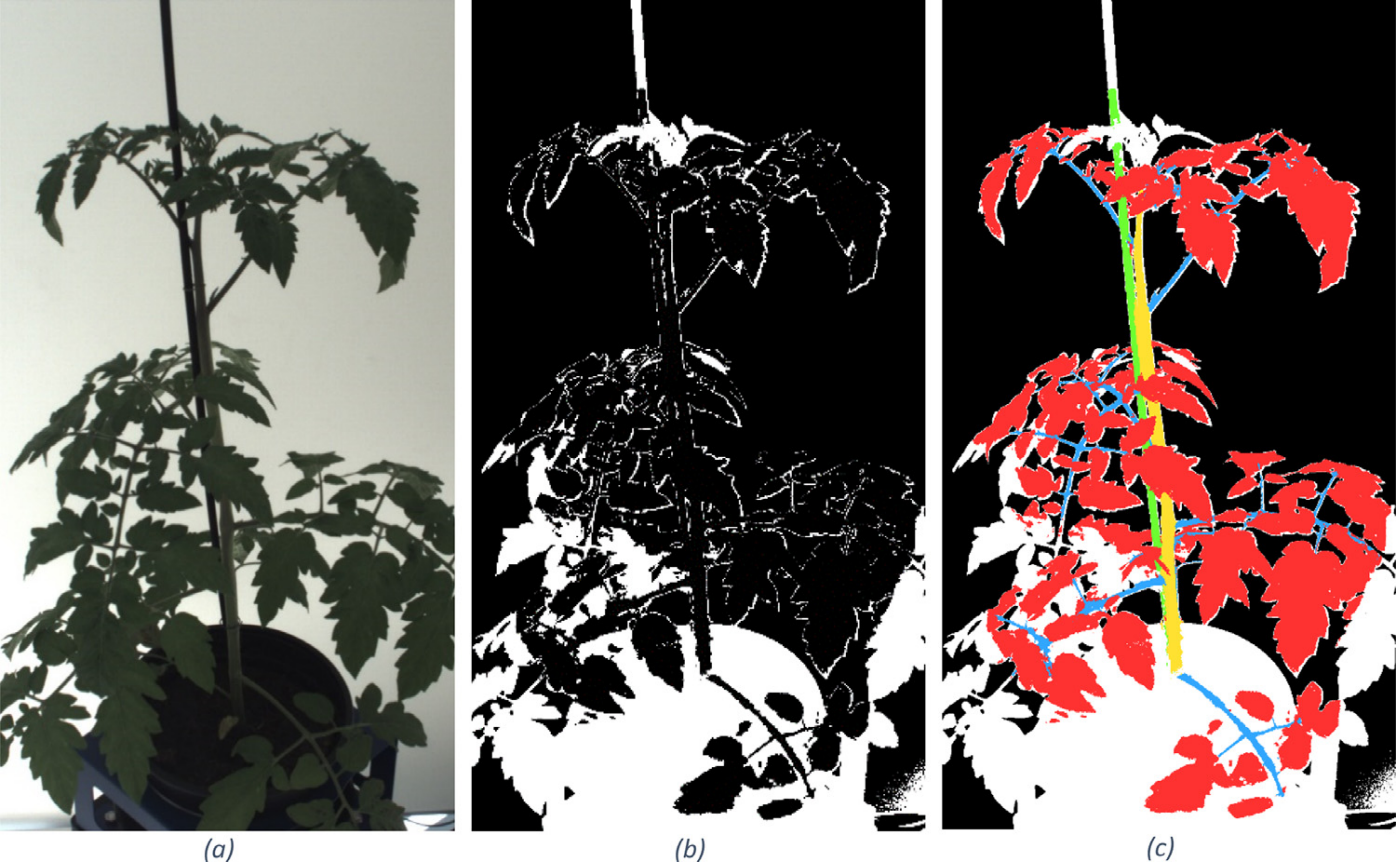
Example image of RGB image (a), corresponding annotated image with binary encoding (b) 0=background 1=leaves, 2=main stem, 3=pole, 4=side stem representing petiole, petiolule and rachis, 255=not annotated, those pixels represent pixels that appear in the 2D image, but not in the 3D point cloud because of limited voxel size, and (c) coloured visualisation of annotation. For more details see section “Annotation of point clouds and images”.

The *images* folder contains all images. For each plant, there is a subfolder with plant_id and fifteen .png files; each file created from a unique camera (cam_id). The filename has a plant id and camera id: {plant_id}_cam_{cam_id).png. The file names of the RGB images correspond with annotated images in the *annotation* folder. In Fig. 2, an example image (a) and annotation (b, c) are visualised.

### 3.3 Point cloud folder

The *point_cloud* folder contains all point clouds. Each plant has a csv file of size Nx9, with N the number of points and nine columns: coordinates (x, y, z)[meters], colour (blue, green, red)[0-255] and normal (nx, ny, nz). Table 2

**Table 2:** Explanation of all headers of the csv files in the point cloud folder.

Header name	Explanation
x	x coordinate of point i
y	y coordinate of point i
z	z coordinate of point i
blue	blue colour value of point i
green	green colour value of point i
red	red colour value of point i
nx	x direction of surface normal of point i
ny	y direction of surface normal of point i
nz	z direction of surface normal of point i

**“ann_versions” folder**

The *ann_versions* folder contains a subfolder specifying annotation version: “0-paper-2Dto3D”. In the future more annotation version can be added. *0-paper-2Dto3D* is subdivided into two subfolders *json* and *annotations*:

├——ann_versions/

**│├——0-paper-2Dto3D/# describing annotation version, currently only 1 version**

**││├——annotations/ # all annotated data**

**││├——jsons/ # jsons with train/val/test split for training the models**

The *annotations* folder has a subfolder for every plant. This folder contains:

- {plant_id}_labels.csv. A csv file with annotated labels consisting of N rows and six columns [Nx6], with N the number of points corresponding to the point cloud and following columns (visualised in Table 3):
  ○ **semantic**: A column with semantic labels: 1=leaves, 2=main stem, 3=pole, 4=side stem, 255=not annotated / noise
  ○ **semantic_with_nodes**: A column with semantic labels including node class: 1=leaves, 2=main stem, 3=pole, 4=side stem, 5=nodes, 255=not annotated / noise. The node class represent points were branching occurs, which could be used to improve robustness of skeletonisation methods.
  ○ **leaf_stem_instances**: A column with index describing leaflet and stem instance
  ○ **leaf_instances**: A column with index describing leaflet instance
  ○ **stem_instances**: A column with index describing stem instance
  ○ **node_instances**: A column with index describing node instance

**Table 3:** Visualisation of point cloud with different annotation labels: (a) semantic, (b) semantic with nodes, the node are hardly visible but shown in purple. (c) leaf stem instances, every unique leaf and stem has unique colour. (d) stem instances and (e) node instances, which are hardly visible due to small size of the nodes.
- {plant_id}_skeleton.csv -> [Mx10], with M the number of vertices, and ten columns: skeleton coordinates (x_skeleton, y_skeleton, z_skeleton)[meters], vertex id with node number (vid), connection to parent node (parent id), edge type to specific the connection between vid and parent id. The connection is either a new leaf/branch, described with “+” or a continuation of previous branch “<”. Four columns with manual measurements: internode length (gt_int_length)[meters], internode diameter (gt_int_diameter)[meters], phyllotactic angle (gt_ph_angle)[degrees] and leaf angle (gt_lf_angle)[degrees]. Those measurements were only done for fourteen plants.
- Fifteen annotated RGB images specified by plant_id and cam_id: {plant_id}_cam_{cam_id}.png.

The ann_version folder also has a subfolder named *json*. In this folder the train, validation, and test jsons can be found with relative path to corresponding point clouds, labels, and skeleton in train validation or test set.

### 3.4 Camera_poses

In the *camera_poses* folder, both intrinsics and extrinsics of each camera are stored in a Json file. The intrinsics correspond with a pinhole camera model. Extrinsics are a transformation matrix in the Open3D coordinate system (right-hand, y-up, z-backward).

## 4 Experimental Design, Materials and Methods

### 4.1 Data acquisition and annotations

#### 4.1.1 Acquiring point clouds

The dataset consist of 44 tomato plants imaged from a running experiment in the greenhouse of the Netherlands Plant Eco-phenotyping Centre (NPEC) in Wageningen. Three distinct cultivars were captured: 19 Brioso, 17 Merlice, and 8 Gardener’s Delight plants. The plants were in the developmental phase, relatively small (<80 cm), and had flowers still in formation. Plants were grown individually in pots and automatically transported to the 3D imaging system.

**Fig. 3:**
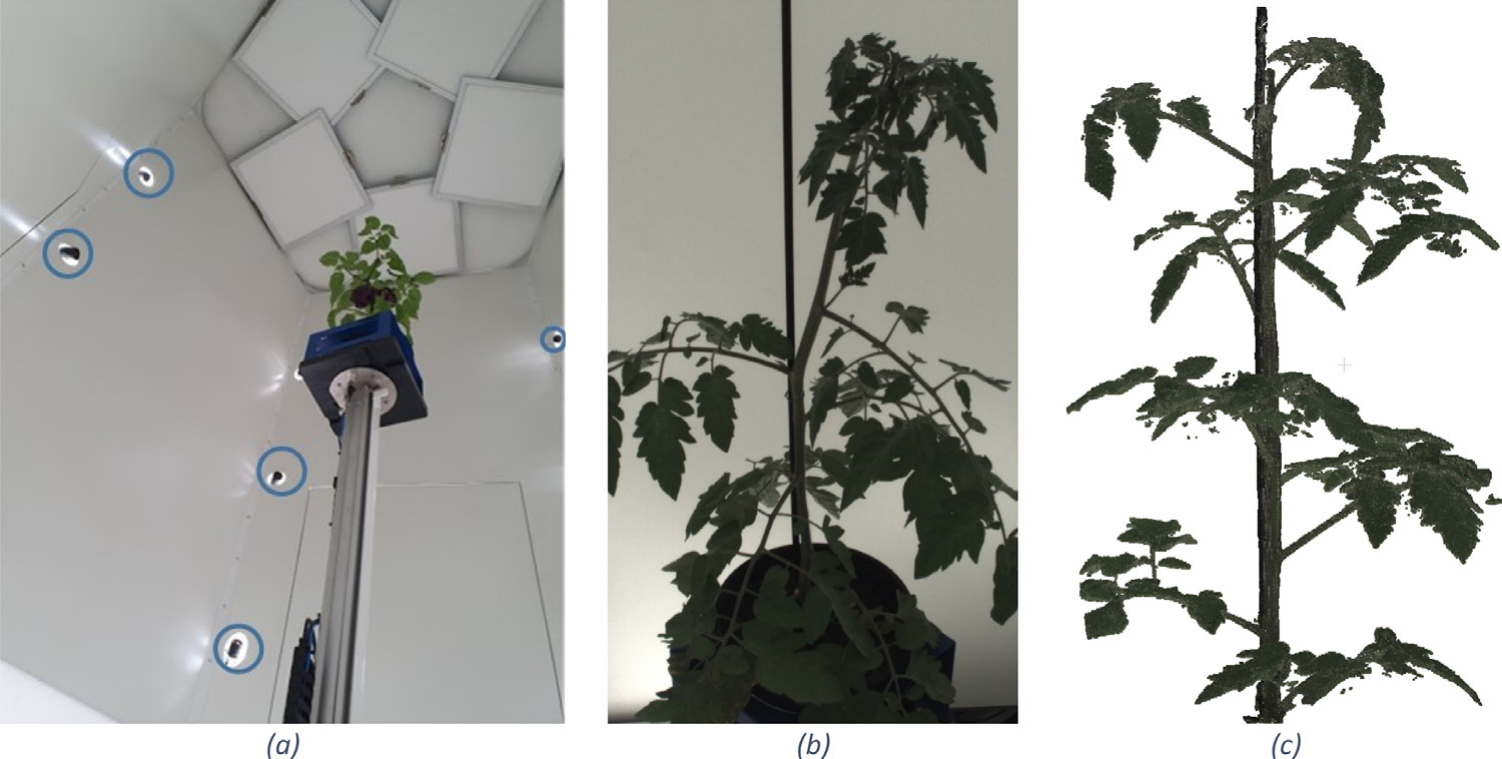
(a) Camera setup to create the point clouds using fifteen cameras on three height levels (only five shown). (b) example RGB image of one of the fifteen cameras. (c) input point cloud of tomato plant with six channels; RGB and surface normals (not visualised). Images adapted from van Marrewijk, van Daalen [2].

The 3D imaging system creates a point cloud using a shape-from-silhouette methodology (Golbach et al., 2016), which leverages lookup tables to produce point clouds within seconds. In contrast, multi-view-stereo or gaussian splatting methods are time-intensive and therefore less suitable for high-throughput phenotyping. The shape-from-silhouette methodology consist of three steps. First, plants were transported to the camera box (Fig. 3a). In this box, the tomato plant was captured with fifteen synchronized cameras (Basler ace Classic, acA1920-25gc) mounted at three different heights levels. At each level, five cameras were evenly arranged around the plant center at an approximate distance of 1.2 meters. Each camera had a resolution of 1080×1920 pixels (width x height) and a 8mm lens with 16${}^{\circ }$ horizontal and 30${}^{\circ }$ vertical FOV. The second step was to segment the plant from the background using an 8-bit RGB colour threshold (R<227∧G<242∧B<122) to get the plant masks per camera. Finally, the shape-from-silhouette methodology initialises a filled voxel space of 400×400×700mm, with a voxel size set to 1mm^3^. Using the calibrated camera intrinsics and extrinsic, the projection of each voxel on the camera images was known. Only voxels that were inside the plant masks of all cameras were marked as occupied and all other voxels were marked empty. The origin of all voxels on the surface of the resulting 3D voxel space were extracted to get the 3D point cloud of the plant as shown in Fig. 3b. The colour values in the point cloud were determined by averaging the colours from all cameras in which the voxel was visible.

#### 4.1.2 Annotation of point clouds and images

The point clouds were manually annotated with CloudCompare into five classes leaves (red), main stem (yellow), pole (green), side stems consisting of petiole, petiolule and rachis (blue) and noise (Fig. 3c).

The annotated point clouds were used to create annotated images by rendering images in Open3D from the known camera poses and intrinsics, which can be found in the *camera_poses* folder. To render images, point clouds were converted to a mesh using the ball-pivoting algorithm in MeshLab with default settings. Some plants were bigger than the voxel space, consequently not all plant pixel in the RGB image could be annotated from the point cloud. This issue was addressed by re-using the original masked RGB images. All pixels that belong to the foreground (plant) but were not annotated using the rendered image were made white. An example image is shown in Fig. 2c. For more details we refer to the publication of van Marrewijk, van Daalen [2] Marrewijk, Xin [10]

[2]**Annotation of skeletons**

**Fig. 4:**
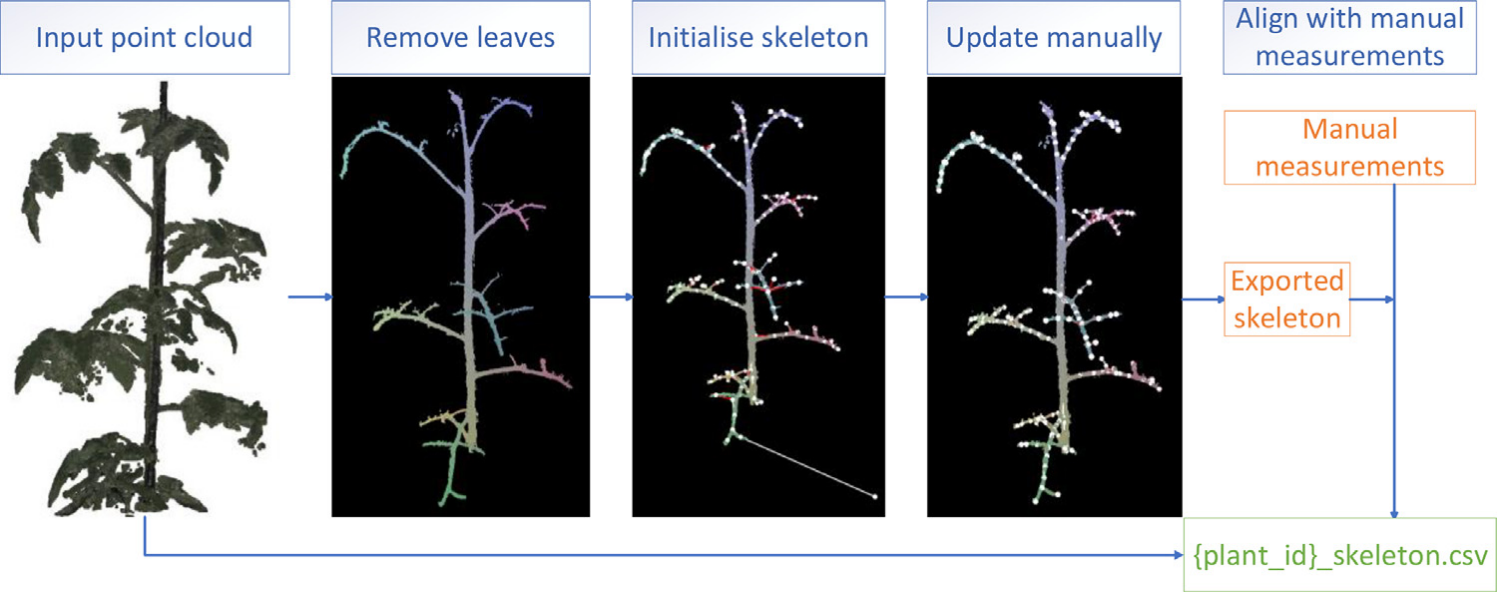
Process to create ground-truth skeletons to create one csv file per plant containing coordinates, colour, labels, surface normal skeleton and manual measurements.

The skeleton consists of nodes, edges describing which nodes are connected, and edge type that classifies whether the connection is a new branch or continuation of previous branch. The nodes and corresponding connections are crucial to extract plant traits like internode length or branching. In Fig. 4, the methodology to create annotated ground-truth skeletons is visualised. Firstly, all points assigned to the leaf class are removed. Secondly, we create a skeleton using the “Xu” skeletonisation algorithm [11] implemented in the PlantScan3D library [12].

The implementation of Xu algorithm in PlantScan3D has six steps:

1. Determine root position of plant. This position was adjusted manually to improve accuracy.
2. Create graph using neighbouring points and assign weights based on Euclidean distance.
3. Traverse graph and find shortest path using Dijkstra algorithm.
4. Cluster points into groups of bins by shortest distance from root to point. The size of the bin was determined automatically by PlantScan3D.
5. Create nodes by calculating centroid of points and create edges by connecting adjacent bins again using Dijkstra algorithm.
6. Assign edge type by traversing all nodes with the root as starting point. The edge type is an important property since it describes whether the current node is a new branch or continuation of current node. The node-order must be correct to be able to determine internode length or leaf angle for example. If the angle between two edges was larger than 60 degrees, then the edge type was classified as new branch. This was denoted with “+” in the csv file.

Due to the automatic clustering of points into bins, centroid calculation, and automatic classification of edge types, some errors may occur. Therefore, individual nodes, edges and edge types are updated manually if needed.

Finally, the skeleton is exported to a csv file in which every node has a vertex id with corresponding position (x, y, z) and edge (parent id). Each node has a parent id, except for the first node also known as “root” position. The exported skeleton is aligned with manual measurements including internode length, leaf angle, and phyllotactic angle. This means that each observation is connected to a vertex id. This connection is crucial to automatically evaluate skeletons and corresponding plant traits.

### 4.2 Manual measurements of plant traits

In this research, manual measurements were taken for fourteen plants of three different varieties Brioso (5), Gardener‘s Delight (3) and Merlice 6). Manual measurements included internode length (m), internode diameter (m), leaf angle (°) and phyllotactic angle (°). Internode length is the length between two plant nodes (insertion points to the stem of either a leaf or a truss) and was measured with a ruler. Internode diameter represents the thickness of the internode in the middle and was measured with a calliper. As the cross-section of internodes is oval-shaped, the diameter was measured at two perpendicular orientations and average value was taken. Leaf angle is the insertion angle of a leaf to the main stem and was measured with a protractor. The phyllotactic angle is the angle between two consecutive organs attached to the main stem (leaves or trusses), in clockwise direction, and was measured with a custom-made protractor [8]. For more details we refer to the paper of [8].

### 4.3 Evaluation methods

An important contribution of this dataset is a comprehensive methodology to evaluate the 3D plant phenotype lines, consisting of the evaluation of:

- Quality of semantic segmentation
- Quality of extracted plant skeleton
- Accuracy of plant-trait extraction

Those three evaluation steps are described in more detail in the sections below. For more details regarding implementation we refer to our GIT: https://github.com/WUR-ABE/TomatoWUR.

### 4.4 Semantic segmentation evaluation

To evaluate the segmentation performance in 2D and 3D following standard deep learning metrics are implemented to evaluate the predicted classes with ground-truth annotations:

- Intersection over Union for each class (IoU_c_). This metric calculates the segmentation performance for each class, using the following equation:
  $IoU_{c}=\frac{TP_{c}}{TP_{c}\;+\;FP_{c}\;+\;FN_{c}}$
- Where TP_c_ is the number of true positive points belonging to class c, FP_c,_ the number of false positives indicating the number of points incorrectly predicted as class c and FN, the number of false negatives, representing the number of points not predicted as class c.
- Macro Intersection of Union (IoU_macro_), a single number representing the average of per-class IoU-values, C is the number of classes. All classes have an equal weight on the metric independent of the number of points in that class.
  $IoU_{macro}=\frac{\sum_{c=1}^{C}IoU_{c}}{C}$
- Micro Intersection of union (IoU_micro_) calculates the performance of all true positives, independent of the class. The IoU_micro_ weights each point equally, dominating classes will resultingly have a larger influence on the metric.
  $IoU_{micro}=\frac{\sum_{c=1}^{C}TP_{c}}{\sum_{c=1}^{C}\left(TP_{c}+FP_{c}+FN_{c}\right)}$

### 4.5 Skeleton evaluation

The skeleton evaluation consists of two evaluation methodologies:

- Plant node evaluation
- Edge evaluation

**Fig. 5:**
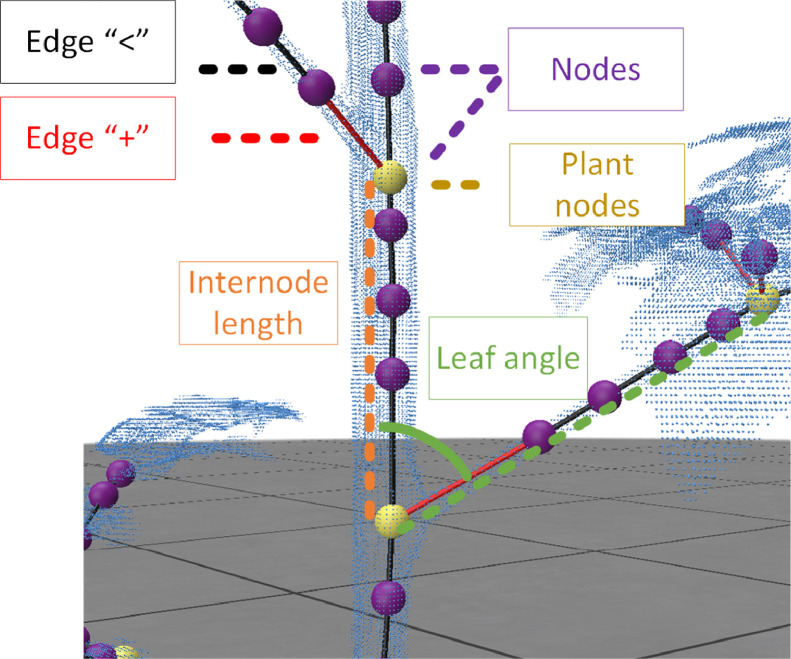
(a) Visualisation of difference between nodes, plant nodes and different edge types. Plant nodes (yellow) contain branches whereas nodes (purple) represent all nodes of the skeleton. Connections are represented by edges, in black indicating continuation of stem and in red a new side branch/leaf.

**Plant node evaluation:** In this evaluation step ground-truth skeletons are compared with predicted skeletons. For the plant node evaluation methodology, a distinction is made between plant nodes and nodes. The difference is highlighted in Fig. 5. Nodes correspond to all nodes in the skeleton, whereas plant nodes are nodes that have a branch indicated in yellow in Fig. 5a. Plant nodes have a biological meaning because they can be used calculate plant traits like internode or leaf and phyllotactic angle. The purple nodes are not strictly necessary for reconstructing the plant architecture. Therefore, in the skeleton evaluation methodology only plant nodes are evaluated.

Our node evaluation methodology is based on Boogaard, van Henten [13]. For every ground-truth plant node, a true positive (TP^node^) is found if there is a predicted plant node within a pre-defined distance threshold (2cm) based on Euclidean Distance. Each predicted plant node can only be assigned to a ground-truth plant node once. If the distance to the nearest predicted node exceeds the distance threshold, the corresponding ground-truth node is considered as a false negative (FN^node^). Likewise, any predicted node not matched with a ground-truth node is classified as a false positive (FP^node^). The TP^node^, FN^node^ and FP^node^ are used to calculate the node precision $\frac{TP^{node}}{TP^{node}+FP^{node}}$ and recall $\frac{TP^{node}}{TP^{node}+FN^{node}}$.

In the precision and recall calculations, all plant nodes are treated equally. However, in practice, nodes of the main stem (0 order nodes) and first order nodes are more vital than second or larger order nodes because these nodes are required to calculate plant traits such as internode length, branching and phyllotactic angle.

**Fig. 6:**
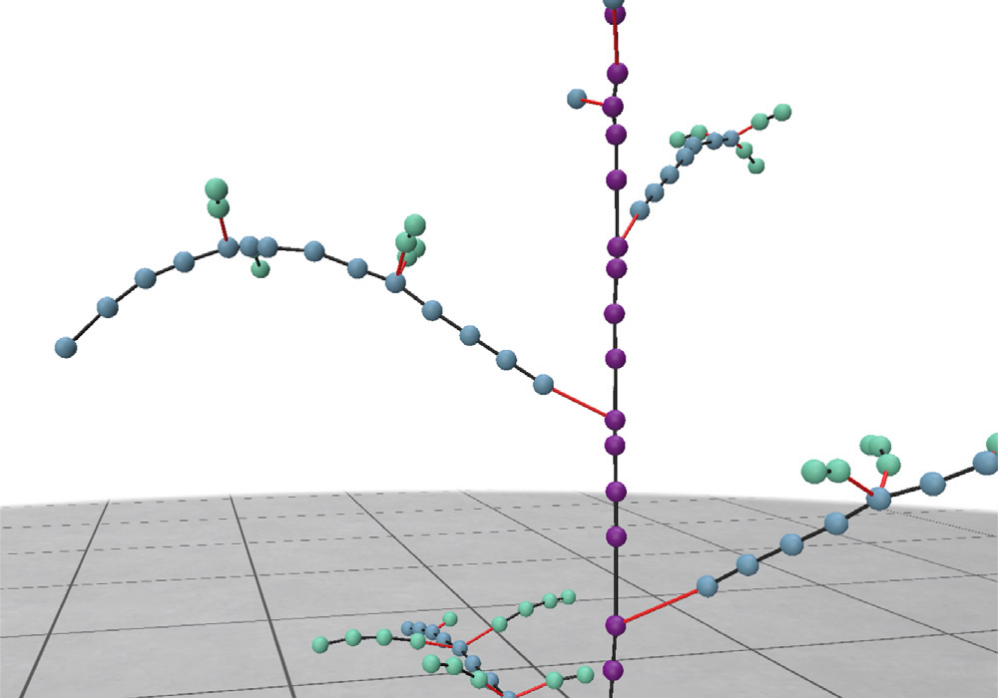
Visualisation of node order, 0 node order = purple, 1 = blue, 2 = light green. The red line represents a new side branch, which increases the node order with +1. For visibility all nodes are shown, in the evaluation methodology only plant nodes are evaluated.

In Fig. 6, the node order is schematically visualised. New branches shown with a red line have a node order of +1 with respect to its parent node. The node importance is included in our evaluation pipeline by indexing only zero, first or second and larger order nodes. For example, to determine the precision and recall for zero order nodes, all zero order nodes of both the ground-truth and predicted skeleton are selected. After indexing, TP^node^, FP^node^ and FN^node^ are calculated as described and converted to node specific precision and recall. This process is repeated for first and second order nodes. In Table 4, the output of the evaluation methodology is shown. Although precision and recall of the “all plant nodes” column is high, precision and recall on especially third node order is low indicate that connection on higher order nodes need to be improved.

**Table 4:** Example of the plant node evaluation.

metric	All plant nodes	0-order node	1-order node	2-order node	3-order node
TP^node^	796	62	198	223	10
FP^node^	237	33	60	141	306
FN^node^	368	72	286	275	38
Precision	0.77	0.65	0.77	0.61	0.03
Recall	0.68	0.46	0.41	0.45	0.21
CD [m]	0.023				

In our node evaluation methodology, the Chamfer Distance (CD) is also included. The Chamfer Distance measures the similarity between two sets of nodes by computing the minimum distance for each all ground-truth (G) and predicted (P) plant nodes. It is not influenced by the distance threshold of 2cm and gives additional insight in the distance between predicted and ground-truth plant nodes. Mathematically, it is defined as:

$$CD\left(G,\;P\right)=\frac{1}{\left|G\right|}\sum_{g\in G}\min_{p\in P}{\left|\left|p-g\right|\right|}_{2}^{2}+\frac{1}{\left|P\right|}\sum_{g\in G}\min_{g\in G}{\left|\left|g-p\right|\right|}_{2}^{2}$$

**Edge evaluation**: The aim of the edge evaluation is to determine whether the connection between the predicted plant nodes is correct. The evaluation method relies on the plant node evaluation method. For every edge in the ground-truth skeleton, check whether both plant nodes of the edge were identified as TP^node^. Next retrieve the corresponding predicted nodes and classify current edge as TP^edge^ if the predicted nodes share the same connection. Otherwise, current edge is considered as a FN^edge^. The number of FP^edge^ is the difference between the total number of predicted edges and TP^edge^. An example of the edge evaluation method is summarised in Table 5.

**Table 5:** Example output of edge evaluation method.

metric	value
TP^edge^	566
FP^edge^	453
FN^edge^	584
Precision	0.56
Recall	0.49

### 4.6 Plant trait evaluation

To evaluate plant traits, the skeleton must be translated to plant traits. In our plant trait evaluation method, we focus on a comparison between predicted and manual measurement of internode length, branching, and phyllotactic angle.

**Fig. 7:**
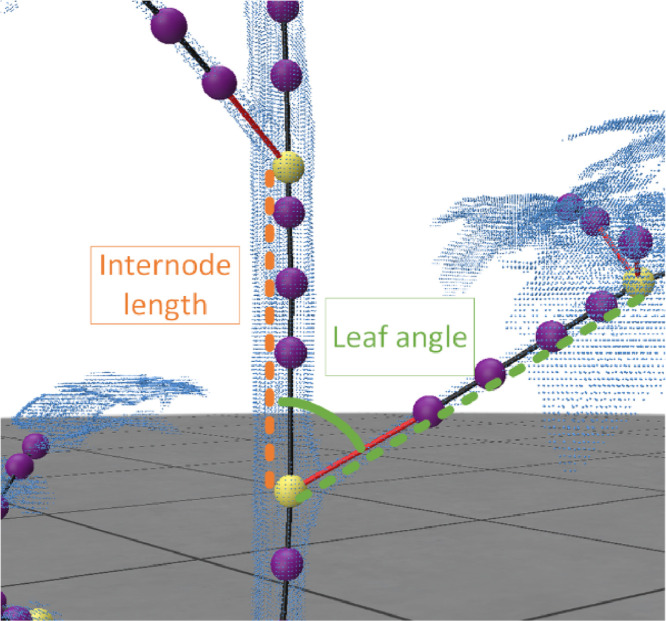
Schematic visualisation of internode length (orange) and leaf angle in green.
- Leaf angle: The leaf angle is the angle of the petiole with respect to the main stem (Fig. 7a). The angle is calculated using the dot product between two vectors a & b from the skeleton. Mathematically, $\theta =\arccos(\frac{a\cdot b}{\left|\left|a\right|∥\left|b\right|\right|})$, where a is the vector from the internode, and b a vector from current plant node to end of petiole. Vector a and b are visualised in Fig. 7a with an orange (a) and green (b) dashed line.
- Phyllotactic angle: the phyllotactic angle is in literature often calculated as the angle between two consecutive branches [8]. In the developed pipeline the angle was calculated by calculating the dot product between the xy-component of vector b (green line in Fig. 7a) and the x-axis of the point cloud [1,0]. The xy-component is derived by eliminating the z-component followed by a vector normalisation.

- Internode length: The internode length is the distance between two plant zero order nodes. In Fig. 7a an example is shown. The orange dashed line is the internode length calculated using the Euclidean distance between two internodes (yellow).

The estimated plant traits in this dataset can be compared with either the ground-truth skeletons, or manual measurements. In our evaluation methodology the error is calculated using the mean absolute error (MAE) and mean absolute percentage error (MAPE), mathematically defined as:

$$MAE=\frac{1}{N}\sum_{i=0}^{N}\left|g_{n}-p_{n}\right|$$

$$MAPE=\;\frac{1}{N}\sum_{i=0}^{N}\frac{\left|g_{n}-p_{n}\right|}{g_{n}}*100\%$$

With g the ground-truth measurements and p predicted plant trait at node n. N refers to the number of TP^node^ that include a ground-truth measurement. Instead of using ground-truth measurements $g_{n}$ can be replaced with plant traits derived from the ground-truth skeleton.

## Limitations

The point clouds in our research were generated using fifteen cameras. However, due to the limited number of cameras and their restricted field of view, some plants exceeded the voxel space. As a result, certain parts of these plants, such as the top portion, may be missing from the point cloud. In addition, some noise is introduced by the shape-from-silhouette methodology since it cannot deal with concavities. Despite this, we believe that the influence on the dataset is limited.

The main contribution of this paper was to create a dataset to evaluate the segmentation, skeletonisation and plant trait extraction step for 3D phenotyping algorithms. The point clouds in this dataset contain fine details and by including skeletons and manual measurements a comprehensive dataset is created. Of course, in the near future we recommend to increase the number of cameras.

Further, it should be noted that in our dataset plants have roughly the same developmental stage and size. This could potentially introduce bias in evaluating 3D phenotyping algorithms. By including three different cultivars, variation in plant structure and leaf arrangement was included. Nevertheless, it is recommended to include more variation in future research to reduce bias.

## Ethics Statement

The authors declare that the ethical requirements of Data in Brief are considered. No human subjects, animals or data from any social media platform was included in this dataset.

## CRediT Author Statement

**Bart M. van Marrewijk:** Conceptualization, methodology, software, validation, data curation, Writing - Original Draft, visualization; **Tim van Daalen:** Data curation, writing review & editing; **Katarína Smolenová:** Data curation, writing review & editing; **Bolai Xin:** Data curation; **Gert Kootstra:** supervision, writing review & editing, conceptualization; **Gerrit Polder:** supervision, writing review & editing.

## Data Availability

4TU.researchdataTomatoWUR: an annotated dataset of 3D tomato plants to quantitatively evaluate segmentation, skeletonisation, and plant trait extraction algorithms for 3D plant phenotyping (Original data).
